# Global monitoring for biodiversity: Uncertainty, risk, and power analyses to support trend change detection

**DOI:** 10.1126/sciadv.adj1448

**Published:** 2024-02-16

**Authors:** Brian Leung, Andrew Gonzalez

**Affiliations:** ^1^ Department of Biology, McGill University, Montreal, Quebec H3A 1B1, Canada.; ^2^ Bieler School of Environment, McGill University, Montreal, Quebec H3A 2A7, Canada.; ^3^ Smithsonian Tropical Research Institute (STRI), P.O. Box 0843-03092, Panama City, Panama.; ^4^ Quebec Centre for Biodiversity Science (QCBS), Montreal, Quebec H3A 1B1, Canada.; ^5^ Group on Earth Observations Biodiversity Observation Network (GEO BON), Montreal, Quebec H3A 1B1, Canada.

## Abstract

Global targets aim to reverse biodiversity declines by 2050 but require knowledge of current trends and future projections under policy intervention. First, given uncertainty in measurement of current trends, we propose a risk framework, considering probability and magnitude of decline. While only 11 of 198 systems analyzed (taxonomic groups by country from the Living Planet Database) showed declining abundance with high certainty, 20% of systems had a 70% chance of strong declines. Society needs to decide acceptable risks of biodiversity loss. Second, we calculated statistical power to detect trend change using ~12,000 populations from 62 systems currently showing strong declines. Current trend uncertainty hinders our ability to assess improvements. Trend change is detectable with high certainty in only 14 systems, even if thousands of populations are sampled, and conservation action reduces net declines to zero immediately, on average. We provide potential solutions to improve monitoring of progress toward biodiversity targets.

## Teaser

Detecting biodiversity improvements is hindered by uncertainty, but solutions exist for monitoring progress to global targets.

## INTRODUCTION

There has never been more interest in allocating resources to reversing biodiversity loss. Human impacts have been detectable on trends in population abundances (*1*–*3*), genetic diversity (*4*), species diversity (*5*, *6*), and ecosystem extent, integrity, and connectivity (*7*). In response, the global community has agreed to the Kunming-Montreal Global Biodiversity Framework [GBF; see Conference of the Parties (COP) decision 15/4] with ambitious targets such as preserving 30% of land and waters by 2030, halting extinctions, maintaining healthy populations, and ensuring the supply of ecosystem benefits to people. The GBF is a theory of change with an explicit focus on addressing the causes of biodiversity loss and the action needed to reverse current biodiversity trends. This requires knowledge of where, why, and how fast biodiversity is changing (*8*). However, the implementation of the GBF is hindered because information about biodiversity change is geographically patchy (*9*), and our estimates of trends in biodiversity and ecosystem metrics are uncertain (*2*).

This uncertainty arises in part because geographic coverage of biodiversity observations is sparse. For example, species occurrences in the Geographic Biodiversity Information System (GBIF) and Ocean Biodiversity Information System (OBIS) cover less than 7% of the world’s surface at 5 km resolution, and less than 1% for most taxa at higher resolutions (*10*). While ~150,000 species have been assessed in the species Red List Index (https://iucnredlist.org/), this remains a small fraction of the global species total. Further, most locations with data have been sampled at only a single point in time, precluding calculation of biodiversity change for these points. Even where populations are monitored over time, uncertainty can be high regarding the true underlying trends due to large within-population fluctuations and differences in growth rates across populations (*2*). It is in the context of this uncertainty that the task of detecting improvements in biodiversity by 2030 under the GBF must be considered.

The monitoring framework of the GBF (see COP decision 15/5) has been established to help countries assess their progress toward the targets by 2030 and achieving the goals by 2050. The indicators of the monitoring framework should allow countries to track their progress across the many dimensions of biological and social transformation prescribed by the targets of the GBF (*11*). The indicators of the monitoring framework measure the amount and type of action implemented to reach the targets for each of the four goals and assess change in direct measures of biodiversity, ecosystem state and extent, and the benefits humans obtain from nature (*12*). The indicators of direct change in facets of biodiversity, such as abundance, require adequate data in the form of time series to reliably estimate trends (*5*, *8*, *9*). The monitoring framework, if properly and equitably resourced, could provide the incentive to fill data gaps that make ready assessment of trends in abundance difficult for large parts of the planet (*10*). However, at this time, we do not know how much more monitoring capacity is needed to obtain accurate and robust estimates of change and to guide future monitoring. The monitoring framework also lacks guidelines on how to address statistical uncertainties in current trends and how to assess progress given this uncertainty. Here, we aim to address these questions, focusing on trends in wildlife abundance for illustration.

Methods for determining whether biodiversity has improved under the GBF must contend with several issues. First, there is substantial variation in the sign and magnitude of trends for different measures of biodiversity. For example, trends in abundance are variable across taxa and scales (*1*). Some taxa are declining rapidly in some regions (*3*), but other taxa are declining weakly, are stable, or are increasing in other regions. Crucially, improvements in one region (e.g., Europe) do not negate declines in others (e.g., Indo-Pacific), although they potentially reflect geographic variation in the drivers of population change and the effectiveness of conservation action (*2*). Second, even within regions and taxonomic groups, populations vary in the magnitude of their trends. Third, even within populations, uncertainty will likely be large given population fluctuations, which are often high (*2*), due to demographic and environmental stochasticity and variable impacts of human drivers. A systematic and statistically sound approach is needed to assess the evidence for improvements in biodiversity trends that accounts for these sources of uncertainty (*8*).

Trend detection is a general statistical challenge in biodiversity monitoring. By detection, we mean the inferential process of identifying that a measure of biodiversity has changed over time relative to a baseline or reference state, with an estimate of the magnitude of change detected along with a statement of statistical confidence (*8*). The uncertainty in time series is a general problem that pervades assessments of change for many biodiversity measures (*9*), including genetic diversity (*4*) and species richness (*13*). Added to these challenges, if we are to evaluate progress under the GBF by 2030, improvements must be detectable within the next decade, and thus, collecting additional long time series will not be feasible.

**Fig. 1.**
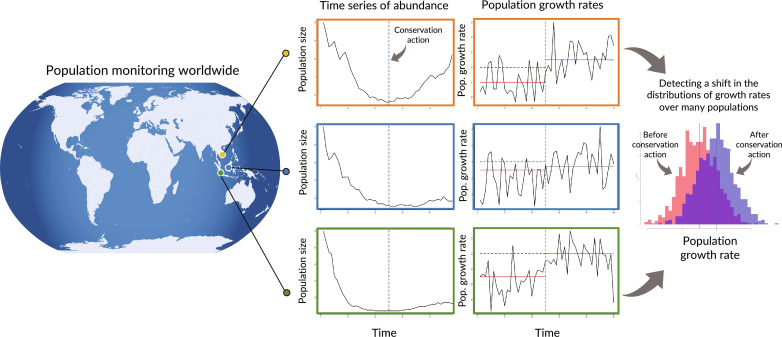
Conceptual diagram of the trend assessment process. Monitoring produces time series of trends in abundance for the many populations worldwide; here, we illustrate this for three populations. Our goals are twofold: (i) to assess trends and the risk of very rapid declines worldwide and (ii) whether we have the statistical power to detect whether population trends have generally improved after conservation action (vertical lines). To do so, we convert population abundance time series (left column of graphs) into annual population growth rate estimates [log(*N*_t+1_/*N*_t_)] (middle column of graphs) to account for the expected temporal autocorrelation. We then estimate the mean trend before (red horizonal lines) and after (blue horizontal lines) implementation of conservation action. We show three populations here, but in reality, each taxonomic group per country (system) is composed of many sampled populations. We aggregate growth rates across all populations within a system, resulting in a distribution of growth rates (right) before (red) and after (blue) action. We tested whether we could detect a shift in the mean of the distribution, before and after conservation.

We have two broad aims: (i) to assess current abundance trends, with a focus on uncertainty. Here, we propose a framing of trend assessments toward a risk-based approach that considers thresholds for both probability (certainty) and rates of decline; (ii) whether future changes in trends can be detected given current uncertainty in the time series across taxa and countries. Here, we propose a “biodiversity-change power analysis” to assess our ability to detect recovery following conservation action. We show that current uncertainty in trends will limit future power, but we provide potential solutions as well. We focus on trends in abundance (Fig. 1), given that it is a direct component of biodiversity (*14*) and underpins a key objective of Goal A of the GBF—to reduce the risk of extinction 10-fold and ensure that the abundance of native wild species is increased to healthy and resilient levels. Our analysis uses the Living Planet Database (LPD), which consists of ~30,000 empirical population trend estimates, and exemplifies the challenges of trend variability and uncertainty described above.

## RESULTS

### Current abundance trends and acceptable risk

Analyses of biodiversity trends often focus on high certainty (e.g., 95% confidence). However, this may not be the best paradigm, given the levels of uncertainty typically associated with population trends, and the potentially severe, irreversible consequences of extinction. Instead, a risk-based framework accounting for severity and its probability of occurrence may be more appropriate to explicitly confront the question of what risks of biodiversity decline we as a society are willing to accept. We analyze current abundance trends for 198 systems (combinations of vertebrate taxonomic class and country) in the context of risk.

**Fig. 2.**
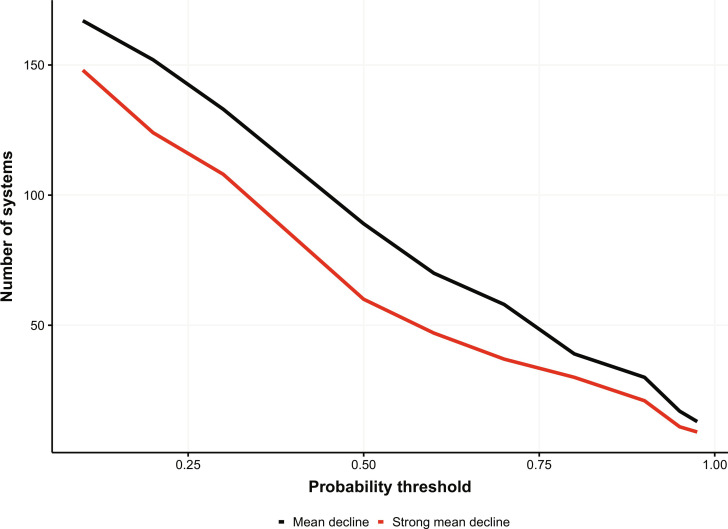
Number of population systems experiencing decline by probability threshold. The number of systems that could be experiencing a decline (black line), or strong decline >1.5%/year (red line), at different probability thresholds. Eleven of 198 systems were identified as declining with 95% certainty (one-tailed credible intervals), while 37 systems had at least a 70% chance of strong decline. The acceptable level of risk (combination of probability and magnitude of decline) remains an open question.

The analysis shows (Fig. 2) that at a 95% credible interval (one-tailed), there are 11 systems that show very high rates of decline (1.5%/year, which, if constant, would result in ~50% abundance decline in a population across 50 years) (table S1). Approximately half of these systems occurred in countries in the Indo-Pacific, but three countries also showed declines in Africa (mammals) and even three European countries (although most strongly increasing systems were also in Europe; table S1). However, we might also consider that a 70% chance of rapid decline is an unacceptably high risk, in which case there would be 37 country/taxonomy systems that would fall into this category (18.7% of the 198 systems examined). At 50% probability, there are 60 systems that could be showing very high rates of decline (30%). Alternatively, as a global community, we might be averse to even a small chance of strong decline, given the potential for sustained declines at this rate to result in the extirpation of many populations. For instance, 148 of 198 systems had at least a 10% probability that they are declining at more than 1.5%/year (Fig. 2).

### Future changes in abundance trends, policy intervention, and biodiversity power analysis

Next, we assess whether we can detect changes in trends due to policy (i.e., actions to reach target 4 of the GBF). We note that there are several ways to analyze change depending on the types of changes we seek to detect [i.e., compare mean abundance, compare trends, or a combination of trends and immediate change (*15*)]. We focus on analyses of trends to see whether conservation actions result in changes in the right direction, where populations are beginning to recover (e.g., perhaps due to habitat restoration). This is less ambitious than full recovery to reference levels but is still a meaningful target given the need to show progress by 2030. In addition, we assess abundance trends, but not trends in natural and human drivers, although this information could reduce uncertainty about the rate and the causes of the trends (*16*, *17*). Nonetheless, the issue of uncertainty and power will likely remain important, and the concepts from this study are generalizable. Last, we do not consider immediate shifts (*15*) (e.g., increases in abundance due to stocking, or reintroduction) and instead focus on changes in conditions.

**Fig. 3.**
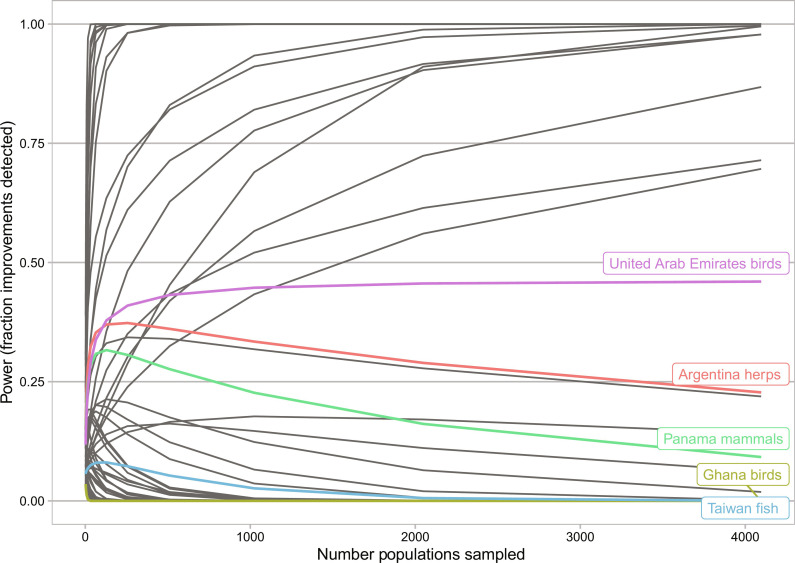
The probability of detecting an improvement. The probability of detecting change after the implementation of conservation action, which changes the mean decline to zero (assuming that current variances of distributions are maintained), is the underlying basis of the biodiversity-change power analysis. The systems where power increases quickly are those that had >95% certainty of decline before action. The others asymptote at zero, i.e., additional sampling improves the resolution of trends post-action, but the ability to detect that an improvement has occurred is limited by the original uncertainty. Each line represents a system (populations separated into taxonomic group by country). We show the 62 systems with estimated mean declines >1.5%/year in the current data from the LPD (see tables S1 and S2 for details). We highlight five systems (using different colors) showing different outcomes discussed in the text (see also Fig. 4).

We focused on 62 systems, which had a current Bayesian posterior mean suggesting strong declines (>1.5%/year), and examined the idealized policy scenario where conservation actions immediately stop populations from declining on average. Even in this optimistic scenario, for 48 systems, we would not be able to reliably detect improved population trends, even with thousands of populations sampled (Fig. 3 and table S2). For 39 systems (more than half), the power to conclude with 95% confidence that populations had improved never exceeded 20% (e.g., fish in Taiwan); moreover, in these cases, power asymptotically declined to zero with increasing number of populations sampled (e.g., mammals in Panama). This result arose because the uncertainty in the estimates of current trends limits our ability to detect changes in trends; with infinite sampling, we can get a very reliable estimate of the future trend, but if the current trends are uncertain, the credible intervals will overlap a mean decline = 0 (the effect of our hypothetical policy). The systems where improvements could be reliably detected were those where the declines were also already highly certain (e.g., fish in Romania).

### Potential solutions

We propose three practical ways to overcome the difficulties in trend change detection.

#### Increased monitoring effort to improve estimates of current trends

**Fig. 4.**
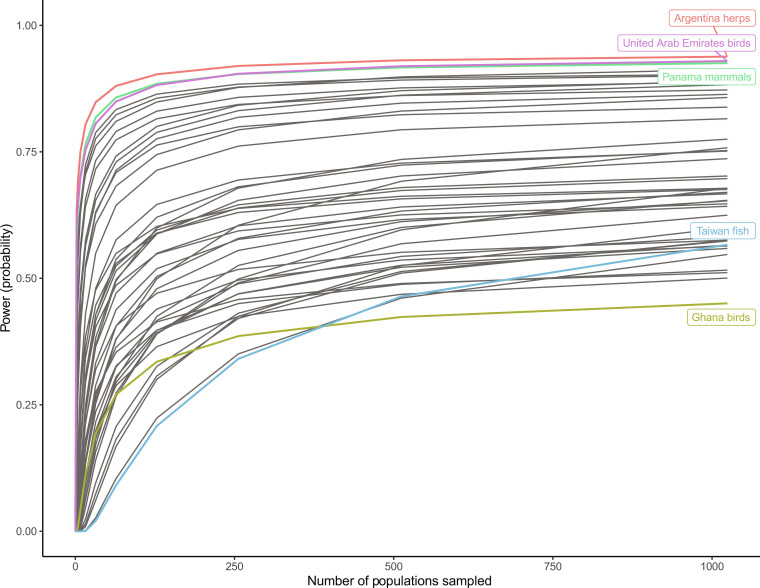
Sampling required to increase power to detect current declines. One potential solution for improving power to detect trend improvements due to conservation action is to reduce uncertainty about the current trends (i.e., improve the baseline for comparison), given that this is the factor limiting trend change assessment. With 250 additional populations sampled, power for most systems levels off. Each line represents a system (country-taxon combination). We highlight five systems (using different colors) showing different patterns discussed in the text (see also Fig. 3). See table S3 for additional details.

The first solution is to improve current trend estimates with additional monitoring, given that the potential power to detect improvements is limited by current uncertainty levels. We focused on the 45 systems with strong declines but high uncertainty (table S3). We asked what level of population monitoring would be required to become 95% certain that these systems were declining on average (Fig. 4 and table S3). For most systems, power levels off with less than 250 populations surveyed. However, for some systems such as fish in Taiwan, power remained low (<40%), requiring >4000 populations to reach >70% power. Other systems such as birds in Ghana had power that remained low (<50%) even with >4000 populations. In contrast, others such as mammals in Panama, birds in the United Arab Emirates, or herps in Argentina could have ~90% power with 250 additional populations sampled. The asymptotic power differed among systems and was dependent on the original posterior distribution, i.e., the portion of the distribution below zero defined the probability that the system was declining, which therefore formed the theoretical asymptote as sampling increased to infinity. Furthermore, portions of the distribution near zero would take increasingly large population sample sizes to report with confidence that they were declining, and thus, the approach to the theoretical asymptote could be slow.

#### Modified threshold for evaluation of conservation outcomes

**Fig. 5.**
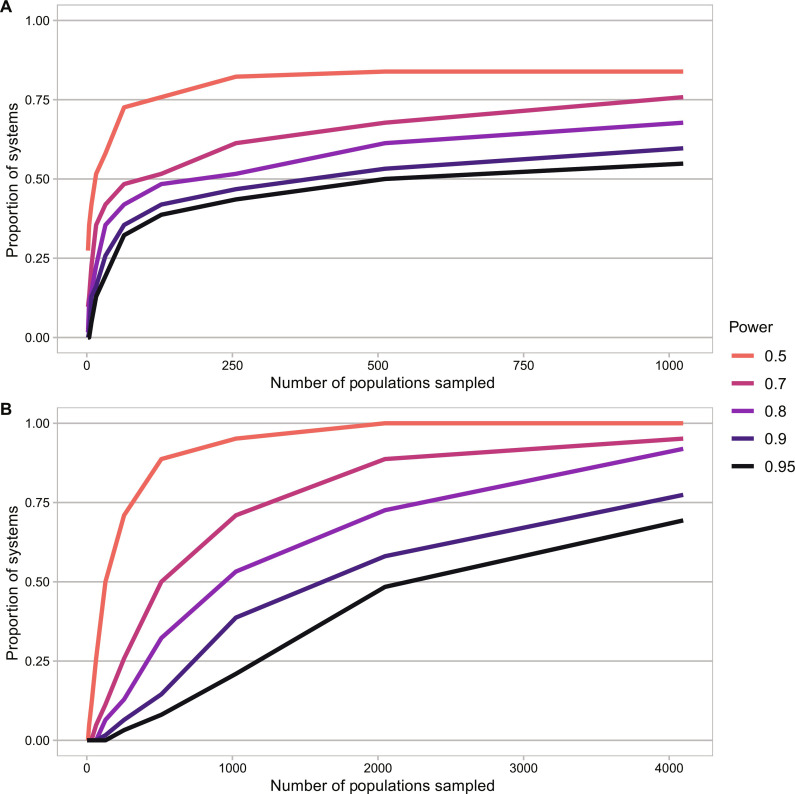
Modified threshold for evaluation of conservation outcomes. Two potential solutions for power analyses to allow trend improvements to be detectable are by (**A**) reducing the threshold of certainty to conclude improvements in trends compared to pre-policy baseline (e.g., using 70% credible intervals) or (**B**) testing post-policy trends against a fixed reference threshold (e.g., 1.5%/year decline). Figure shows the fraction of the 62 systems analyzed, which reached a given level of power (different colored lines), as the number of populations sampled increases (see table S4 for details).

A second way to address uncertainty is to allow the detection of trend improvements by simply accepting a lower threshold of certainty. For instance, if we set our threshold of certainty to 70% that conservation action has resulted in an improvement in population trends, we could detect improvements in roughly half (32) of the country/taxon systems with 80% power with ~250 populations sampled, and 49 of 62 systems had >80% power when 4000 populations are sampled. However, 10 country/taxon systems still had power less than 50%, with some declining to zero (Fig. 5A and table S4). Again, this result reflects the high initial uncertainty, driven in part by the low number of currently sampled populations.

#### Setting a “reference” threshold for evaluation of conservation outcomes

Last, a third practical solution is to compare post-policy trends to a reference threshold of decline, rather than establishing whether improvements in the trend have occurred. In this case, power would no longer be limited by current levels of uncertainty. For a concrete example, we may want to determine the power to be 70% certain that populations are doing better than a threshold of 1.5%/year mean decline, given that a policy reduces the true mean decline to zero (Fig. 5B). We find that we would be able to eventually detect that populations met this criterion (in contrast to Fig. 3), although in some systems, it would require thousands of populations to be sampled. For instance, given their high background levels of variation, mammals and birds in Australia and fish in Taiwan and Romania had the lowest power (<71%), even with 4000 populations sampled (table S4). In contrast, these systems all had >90% power with option 2 (i.e., comparing against the current trend, with 70% certainty that an improvement had occurred), because the current empirical decline in those systems is much greater than the reference threshold used of 1.5%/year. These results illustrate that different options may be optimal for different systems.

## DISCUSSION

The GBF is built on a theory of change articulating the implementation of the knowledge and action needed to reverse current biodiversity trends and result in society living in harmony with nature by 2050. Our analyses of statistical power to detect change in population trends suggests that in many countries and for certain taxa we may not be able to tell whether our conservation actions are achieving the reversal of declining trends desired [“bending the curve,” (*18*)] by the goals and targets of the GBF. This uncertainty arises because of the high variability in trends estimated to date and our incomplete knowledge of these trends given current available data. There is no immediate fix for this background level of uncertainty, but the international community can invest in monitoring to assess current trends and rates of change to guide progress toward the goals of the GBF between now and 2030 and 2050.

The monitoring framework of the GBF guides countries with the task of acquiring the data and calculating the indicators needed to assess their progress toward the goals arising from their actions across the 23 targets. We show that while data and an indicator of trend change are necessary, they are not sufficient to establish the degree of progress toward the goals. The monitoring framework must offer guidelines on how countries can establish with confidence that biodiversity change is occurring because of conservation action and doing so fast enough to meet the targets set for the goal.

### Four recommendations for the monitoring framework

The text of monitoring framework (paragraph 7, COP decision 15/5) invites organizations, including the Group on Earth Observations Biodiversity Observation Network, to support the operationalization of the monitoring framework for the Kunming-Montreal GBF. We thus propose four options to inform the operationalization of the monitoring framework and to help countries assess their progress by finding ways to overcome the constraints imposed by current data availability. This guidance is focused on the two main objectives of this study: to (i) assess current abundance trends, with a focus on uncertainty (i.e., given existing data), and (ii) assess whether we are likely to be able to detect future changes in trends (i.e., setting targets before data collection). The first recommendation corresponds to objective 1, and the next three recommendations are context dependent but provide potential solutions for objective 2, given our finding that it may be difficult to detect improvements after conservation action.

#### Interpret trends using a risk framework

We propose the adoption of a risk framework. While only 11 systems were severely declining with 95% certainty, given the potential ramifications of widespread biodiversity loss, it seems appropriate, under the precautionary principle (*19*), to also consider lower probabilities of certainty. This should encourage an explicit discussion of acceptable levels of risk that parties to the GBF are willing to accept. This will have implications for the strategic investment in resources (cost and efficacy) dedicated to recovery of species and the protection and restoration of their habitats. Biodiversity science can implement criteria like those used in climate science to evaluate the weight of evidence for detecting and attributing climate trends and the levels of acceptable risk associated with trends estimates (in) consistent with policy outcomes (*8*, *20*).

#### Improve current trend estimates with additional monitoring

There is great value in allocating resources to continue monitoring “status quo” trends (i.e., populations not benefiting from GBF interventions), given that current levels of uncertainty are a key limiting factor to evaluate our progress toward biodiversity goals. Because we also want to implement biodiversity strategies as soon as possible, a monitoring framework could entail “experiments,” wherein some populations are managed under status quo conditions and monitored, thereby increasing the sample size and reducing uncertainty of current trends, while management intervention improves the condition of populations in the remainder of the system (to maximize the probability of achieving stated goals). An example that this approach can be effective is the large-scale construction of hundreds of new ponds that halted and reversed the decline in amphibian populations, including Red-Listed species, in Switzerland (*21*).

#### Modify threshold for evaluation of conservation outcomes

We argue that it may be reasonable to set a lower certainty threshold to assess whether conservation action has resulted in trend improvements. Our results suggest that in many systems, we would not be able to conclude with 95% certainty that abundance trends have improved. However, it could be justifiable to conclude that our goals have (likely) been met if there is, for instance, a 70% probability that biodiversity has improved. Simply put, the quantitative outcomes for biodiversity that are chosen must be assessable to be valid, and these criteria should be stated a priori. This is required for the application of an effective theory of change.

#### Use an absolute reference threshold for evaluation of conservation outcomes

A reference threshold of decline may be useful to be considered as a metric for evaluation. In this case, evaluation would not be affected by current levels of uncertainty; we would not be able to conclude that policies have resulted in any improvements but can at least conclude that systems are not declining beyond a certain rate. However, the number of populations required to conclude that even catastrophic declines are not occurring can still be large.

The choice from the mix of options we suggest will likely depend on the context. Some systems that are declining with high certainty would only require sufficient post-policy sampling to conclude that improvements are occurring. Others could show higher certainty in current trends with modest additional sampling, which would thus make it possible to detect improvements post-policy as well. Still, other systems could benefit from a mixture of strategies, improving current trends and requiring lower certainty to conclude success. For systems with very high uncertainty, one could switch to comparisons against a fixed reference threshold of decline. Last, while not analyzed here, additional predictors of decline could reduce uncertainty about trends and increase power. However, putative drivers of biodiversity loss do not always have the expected effects (*22*) and can often interact over large geographic scales or have lagged effects (e.g., because of historical land-use legacies) on population trends (*23*). Regardless, we suggest jointly monitoring drivers and populations over time in a detection and attribution framework for biodiversity monitoring (*8*).

### A GBiOS for reliable trend detection

While we present our analysis using real data from the LPD, these populations are likely a biased sample of change worldwide, which could affect estimated patterns of change over time for any given system. The exact consequences of this potential bias are generally unknown. More broadly, given that monitoring will likely always represent a sparse sample of the total geographical space, there is a danger that monitoring sites are not representative of where conservation actions are occurring. This could underestimate the overall change resulting from management, or perversely, one might focus conservation actions only on the locations that are monitored while assuming that actions are system-wide (thereby overestimating the overall benefit). Thus, while monitoring is critical to ensure that real progress is being made toward goals, it must be done carefully and rigorously.

A global biodiversity observing system [GBiOS; (*24*)] collecting data across a network of monitoring sites worldwide could be designed to fill the geographic and taxonomic gaps in our knowledge of biodiversity change and reduce uncertainties over time. Further, systematic sampling in GBiOS could help ensure that observed trends are representative of overall biodiversity changes.

Properly specified with assessable targets, an effective GBiOS would generate the data over a representative network of sites at the frequency and geographic scales needed to detect changes in population trends [and trends in other essential biodiversity variables, (*25*)] soon after they have begun to respond to conservation action. Monitoring by a GBiOS could follow the dual approach we suggest, with experiments managing some populations under status quo conditions to reduce uncertainty of current trends while implementing policy interventions for the remainder of the system (to maximize the probability of achieving stated goals). This approach would provide insight into how many populations need to be monitored under the status quo and establish the management effort needed to reach acceptable levels of recovery and certainty.

In this study, we assumed an idealized case where conservation action was effective immediately, and we found that detection would be difficult even in this scenario. The minimum time to detect change in population trends of a given magnitude is a particularly important issue for global biodiversity policy (*26*). If we are to succeed in the objectives of the GBF to reverse biodiversity decline, rigorous, systematic sampling, with full consideration of uncertainty and trend detectability, is crucial to determine whether our policies are having the intended effects or whether reorientation is necessary. The monitoring of biodiversity trends would be well supported by the establishment of a GBiOS designed explicitly to assess and guide progress to targets under uncertainty (*24*).

In summary, we addressed the problem of how uncertainty in our knowledge of existing trends in monitored populations constrains our ability to assess whether conservations goals will be achieved globally in the coming decades. For the case of global trends in population abundances, this is the probability of achieving a reduction in the average rate of decline for the global distribution of threatened populations, at an agreed upon point in the future (e.g., by 2030 or 2050). We show that these goals should be considered carefully in the context of trend detection, given the uncertainty we have about current global average rate of declines. We provided four ways to make analyses more feasible. Below the global level, the same definition can be applied to rates of population change disaggregated to reflect progress country by country and taxon by taxon. Given that the effort and resources that will be invested in conservation will exceed hundreds of billions of dollars worldwide, it is important that we know whether our actions have been effective or, alternatively, when we should adapt our strategies to ensure that we are on course to achieve our biodiversity goals.

## MATERIALS AND METHODS

### A power analysis for trend detection

The probability of achieving a policy goal, given the environmental state of the world and the current sampling of populations, defines the efficacy of the conservation action. Our ability to detect progress with a given level of certainty is defined by a statistical power analysis.

Power analysis for biodiversity trend detection has at least two scales—a local scale for trend change detection within a site and then at a regional (or global) scale where we assess the power to detect change in the aggregate distribution of trends across many sites or groups of populations. The task of detecting the change in any varying measure of biodiversity can therefore be characterized as a hierarchical statistical problem. We have many local sites exhibiting fluctuations and trends over time that will vary from site to site because of heterogeneous effects of natural and human drivers. The statistical power will vary with the biodiversity indicator, the type of change we are looking for within and across populations, and the effect size of the conservation action.

Statistical power may be increased by reducing measurement error, increasing sample size (i.e., the length of time series or number of populations), or increasing the strength of conservation measures. We focus on calculating the sample size (i.e., the number of populations monitored and for which trends are estimated), assuming that sampling methods will reflect historical levels of accuracy, and additional sampling of populations will be constrained by the maximum 7-year window before 2030. We make the simplifying scenario that conservation measures have successfully reduced the true mean rate of change to zero, but any other level of efficacy of conservation action could be used as well.

### Estimates of current population trends

We analyzed population time-series data, by using the LPD (http://livingplanetindex.org/data_portal). While the approaches we will discuss are applicable to any group of population trends, the LPD is arguably one of the most extensive compilations of time-series data publicly available, currently comprising >30,000 vertebrate populations from 1970 to 2018, and it illustrates many of the challenges associated with detecting improvements in trends. We focused on country-level analyses, given that goals of the GBF will be based on national-level reporting. We further broke analyses down by taxonomic class (fish: Actinopterygii, Elasmobranchii, Holocephali, Myxini, Chondrichthyes, Sarcopterygii, and Cephalaspidomorphi; birds: Aves; mammals: Mammalia; and herpetofauna: Amphibia and Reptilia, henceforth, termed "herps"), resulting in 488 country-taxa combinations (henceforth, termed “system”). We restricted our analyses to systems with greater than 10 populations sampled, resulting in 198 systems.

We focused our analyses on general trends in each system and used the Bayesian hierarchical mixture model (BHM) to remove the extreme declining (and increasing) trends, which have been shown to comprise only a small fraction of populations (1.4%) but could have a pronounced effect (*2*). In the context of policy evaluation, a few extremes could mask detection of general improvements or, conversely, could result in apparent improvements even as most populations continue to decline. In addition, a Bayesian hierarchical approach also provides a measure of uncertainty as an outcome of analyses via the posterior distribution, accounting for intra-population fluctuations as well as inter-population differences in growth rates, and differences in the number of data points comprising the time series. Arguably, systems undergoing widespread declines are the most concerning, and thus, we focus on systems where the mean system-wide annual rate of decline was greater than 1.5%/year (if all populations continued at that rate, 50% would be lost over 50 years).

We define parameters θ and τ as the system-wide mean and SD of log growth rates (after removing the extremes using the BHM), respectively, and σ was the SD of within-population fluctuations. Thus, we can specify the power problem in terms of whether improvements in the system-wide mean log growth rate (θ) would be detectable under the scenario where management actions reduced the true mean decline to zero (Fig. 1).

### Risk assessment framework

Given the data from the LPD, there are high levels of uncertainty about the trends in the time series (based on 95% credible intervals), but this does not preclude the possibility that strong declines are occurring. Here, we highlight risk assessment as a useful framework, given its consistency with how we treat other disasters, and its natural relationship with Bayesian statistics. A risk framework accounts for both the severity of population decline and the probability (*P*) we are willing to accept that these trends are occurring.

We estimate the probability *P* by taking the proportion of the posterior distribution of θ with values less than some threshold of decline *T*. We considered both systemic declining trends (*T* = 0) and severe declines (set at beyond 1.5%/year, *T* = −0.015).

### Effect of a biodiversity monitoring system

We begin with a model of how additional data will improve the certainty about current trends, because this is the more complete formulation; analyses of changes in trends (e.g., due to policy) is a special case of this model. We start with the posterior distributions based on historical data, which give us the probability that each parameter set [θ and τ, for mean and SD of log (growth rates) between populations, respectively] is “true,” from which all else can be derived. This allows us to do two things: (i) The posterior distribution acts as the new prior distribution (i.e., Bayesian updating) in the presence of new data, and (ii) while the true parameters are unknown, we know how probable they are from the original posterior distributions, and we know the distribution of additional data that would be generated for each possible parameter set (θ and τ).

We focus on the mean growth rates of a system (θ) and begin with the observed posterior distribution, taking the mean theta (${\bar{\theta }}_{1}$) and SD of theta (*s*_1_), across all Markov chain Monte Carlo (MCMC) runs. This will become the prior, as we “collect” new data. We need to then determine what new data are likely to be collected and the updated (posterior) distribution, which is the outcome of the new data and the “Prior” distribution (${\bar{\theta }}_{1}$ and *s*_1_). This requires several steps: The new data that are collected will be determined by the true generating distribution (which we can estimate up to a probability). Thus, we begin with each MCMC realization i (probability of “reality” as defined by θ*_i_* and τ*_i_*, for mean and SD of growth rates between populations, respectively). Second, each parameter set would theoretically result in a distribution of potential observations, in this case, the sample mean of growth rates ($\bar{x}$), given the number of populations (*p*) and number of years (*n*) sampled for each population. Third, we calculate the effect of each value of $\bar{x}$ on the posterior distribution (denoted using subscript 2; i.e., ${\bar{\theta }}_{2}$ and *s*_2_), thereby updating the original estimates ${\bar{\theta }}_{1}$ and *s*_1_. Fourth, for each realization ($\bar{x}$), we calculate the credible intervals for the generated posterior distribution (${\bar{\theta }}_{2}$ and *s*_2_) and determine whether it exceeds some threshold (*P*) to conclude that a change has occurred beyond some threshold rate (*T*) (e.g., mean rate of decline is more than 1.5%/year). Fifth, we normalize by the relative probabilities of occurrence of $\bar{x}$, θ, and τ, to obtain an overall estimate of power, given all possible realizations. The exact formulation is as follows:

First, for each given reality (MCMC run *i*, generating θ*_i_* and τ*_i_*), the variance of new data (*s*^2^_0,*i*_) would be determined by the among-population variance (τ*_i_*) and within population variance (σ^2^). The distribution of sample means has

$$s_{0,i}^{2}=\frac{\tau_{i}^{2}+\frac{\sigma^{2}}{n}}{p}$$ (1)

where *p* is the number of populations and *n* is the number of samples within populations. Given this variance $s_{0,i}^{2}$, we can determine the probability of new sample means ($\bar{x}$_*i*,*j*_) for each parameter set θ*_i_* and τ*_i_*, which would be generated following the distribution

$${\bar{x}}_{i,j}∽N\left(\theta_{i},s_{0,i}^{}\right)$$ (2)

In the next step, for each realization ${\bar{x}}_{i,j}$, we can analytically calculate the updated posterior distribution for θ_*i*,*j*_, which has a variance

$$s_{2,i}^{2}=\frac{1}{\frac{1}{s_{1}^{2}}+\frac{1}{s_{0,i}^{2}}}$$ (3)

As sample size increases, $s_{0,i}^{2}$ becomes smaller and the new samples dominate the signal; at its limit, $s_{2,i}^{2}=s_{0,i}^{2}$. When no additional samples have been collected, $s_{2,i}^{2}=s_{1}^{2}$

and mean

$${\bar{\theta }}_{2,i,j}=s_{2,i}^{2}\left(\frac{{\bar{\theta }}_{1}}{s_{1}^{2}}+\frac{{\bar{x}}_{i,j}}{s_{0,i}^{2}}\right)$$ (4)

With these two values, we can then determine the probability (i.e., the proportion of the posterior distribution) beyond a threshold *T*.

$$Z_{i,j}=\frac{T-{\bar{\theta }}_{2,i,j}}{s_{2,i}^{}}$$ (5)

where *Z* is the standard normal deviate, and *Pr*(*Zi*,*j*) is the fraction of the posterior distribution less than *T*.

Last, we calculate the overall chance of observing a decline (*D*) across all possible scenarios by integrating across all values of $\bar{x}$, θ, and τ.

$$D=\int \int \int f\left(\bar{x}|\theta ,\tau \right)\text{pr}\left(\theta ,\tau \right)*\left\{\begin{array}{c} 1\;\mathrm{if}\;\text{Pr}\left(Z\right)\geq P\\ 0\;\mathrm{if}\;\text{Pr}\left(Z\right)<P \end{array}\right.d\bar{x}d\theta d\tau$$ (6)

where $f\left(\bar{x}|\theta ,\tau \right)$ is the normalized probability density for observing $\bar{x}$, and *P* is a threshold probability (analogous to a credible interval) of being worse than the threshold decline of *T* (e.g., if *T* = 0, and *P* = 0.95, which would indicate that there was a 95% chance that the trend was declining).

### Detecting improvements compared to current trends

We can modify the model above and conceptualize detecting change as an additional parameter (θ*_d_*) for data collected after a policy intervention. Specifically, if policy results in an improvement, θ*_d_* should be greater than zero, with a certainty based on the credible intervals (e.g., a one-tailed 95% credible interval). In notation, the likelihood across all populations could be described as

$$L=\prod N[\bar{x}|\left(\theta_{1}+d\theta_{d}\right),s]$$ (7)

where *d* is a dummy variable: *d* = 1 corresponds to new data, *d* = 0 corresponds to the original data, and *s* is the SD of $\bar{x}$.

For power analyses, we can use Eqs. 1 and 2 again from the posterior distribution to determine the new data that would be generated probabilistically, except that we replace θ*_i_* in Eq. 2 with the “alternative hypothesis” value (*A*) (i.e., effect of the given policy). We assume that the variation remains unchanged using Eq. 1. In notation

$${\bar{x}}_{i,j}∽N\left(A,s_{0,i}^{2}\right)$$ (8)

Since data points are independent, it is equivalent to combining two independent likelihoods. Thus, the posterior distribution of θ_1_ would be the same as from the original pre-policy data, and the posterior distribution of θ_2,*i*,*j*_ would be the same as if it were calculated only for the new data (${\bar{x}}_{i,j}$). For each simulated sample mean (Eq. 8), using a flat prior, the mean (θ_2,*i*,*j*_) and variance ($s_{2,i}^{2}$) of the posterior distribution would be

$$\theta_{2,i,j}={\bar{x}}_{i,j}$$ (9)

$$s_{2,i}^{2}=\frac{\tau_{i}^{2}+\frac{\sigma^{2}}{n}}{p}$$ (10)

Thus, to determine the probability of detecting an improvement compared to the current population trends, we need to determine the distribution of θ*_d_*, which can also be calculated using the distribution of differences: For each ${\bar{x}}_{i,j}$, the mean of the posterior distribution of differences would be

$${\bar{\theta }}_{d,i,j}=\theta_{2,i,j}-{\bar{\theta }}_{1}$$ (11)

and the posterior variance of θ*_d_* would be the sum of the individual variances

$$s_{\theta_{d},i}^{2}=s_{\theta 1}^{2}+s_{2,i}^{2}$$ (12)

The credible intervals become simple to calculate then, and will follow a *Z* distribution

$$Z_{i,j}=\frac{\theta_{d,i,j}}{s_{\theta_{d},i}^{}}$$ (13)

From this, we can calculate the probability of detecting an improvement at a threshold of confidence *P* by integrating across the posterior distribution of variances (τ) used to generate samples (the alternative mean *A* was chosen and constant), and the distribution of realizations ($\bar{x}|\tau$). In notation

$$\Phi =\int \int f\left(\bar{x}|\tau \right)\text{pr}\left(\tau \right)*\left\{\begin{array}{c} 1\;\mathrm{if}\;\text{Pr}\left(Z\right)\geq P\\ 0\;\mathrm{if}\;\text{Pr}\left(Z\right)<P \end{array}\right.d\bar{x}d\tau$$ (14)

where Φ indicates the power to detect improvements and is dependent on the choice of the alternative mean *A*, the number of populations *p*, the number of estimates per population *n*, the within-population fluctuations, the posterior distribution of variances τ, and the pre-policy posterior distribution of means θ_1_.

### Detecting improvements compared to a reference threshold of decline

As a final analysis, we calculated the probability of detecting that the trends after policy (*A*) are better than some prespecified reference threshold level of decline (*T*). In this case, we do not need to consider the uncertainty in θ_1_ within the current trends (but we still use the posterior distribution of τ to generate values of between-population variance, Eq. 8) and can focus solely on the distribution of new data (Eqs. 9 and 10).

$$Z_{i,j}=\frac{\theta_{2,i,j}-T}{s_{2,i}}$$ (15)

We replace Eq. 13 with Eq. 15 and evaluate using Eq. 14.
