# Supplementary material for: Global monitoring for biodiversity: Uncertainty, risk, and power analyses to support trend change detection
Source: Sci Adv. 2024 Feb 16;10(7):eadj1448. doi: 10.1126/sciadv.adj1448 (PMC11639671; doi:10.1126/sciadv.adj1448)
Supplement: Supplementary file 1 — Tables S1 to S4 [file sciadv.adj1448_sm.pdf]

Science Advances
AAAS

# Supplementary Materials for

## Global monitoring for biodiversity: Uncertainty, risk, and power analyses to support trend change detection

Brian Leung and Andrew Gonzalez

Corresponding author: Brian Leung, brian.leung2@mcgill.ca

*Sci. Adv.* **10**, eadj1448 (2024)
DOI: 10.1126/sciadv.adj1448

**This PDF file includes:**

Tables S1 to S4

Table S1: System Risks

| System | Theta (sd) | N | P(Theta) < 1.5 | P(Theta) < 0 |
|---|---|---|---|---|
| Romania Fish | -0.83 (0.331) | 53 | 0.9930000* | 0.9938235* |
| Pakistan Birds | -0.505 (0.103) | 59 | 1.0000000* | 1.0000000* |
| India Birds | -0.41 (0.08) | 187 | 1.0000000* | 1.0000000* |
| Nigeria Birds | -0.385 (0.29) | 17 | 0.9004706 | 0.9084902 |
| Portugal Herps | -0.38 (0.136) | 15 | 0.9944510* | 0.9956275* |
| Argentina Herps | -0.366 (0.227) | 29 | 0.9403529 | 0.9476667 |
| Albania Birds | -0.305 (0.176) | 13 | 0.9531765* | 0.9610392* |
| Croatia Birds | -0.256 (0.16) | 14 | 0.9358627 | 0.9467255 |
| Iceland Mammals | -0.239 (0.17) | 16 | 0.9095490 | 0.9241373 |
| Guam Birds | -0.209 (0.064) | 15 | 0.9986078* | 0.9994510* |
| Puerto Rico Fish | -0.197 (0.144) | 37 | 0.8977451 | 0.9149216 |
| Philippines Fish | -0.194 (0.143) | 59 | 0.8944118 | 0.9130196 |
| Panama Mammals | -0.194 (0.123) | 13 | 0.9323922 | 0.9464706 |
| Germany Fish | -0.145 (0.081) | 62 | 0.9495490 | 0.9663529* |
| Central African Republic Mammals | -0.13 (0.042) | 20 | 0.9949804* | 0.9974706* |
| China Fish | -0.128 (0.054) | 15 | 0.9818824* | 0.9908627* |
| Cte D'Ivoire Fish | -0.108 (0.161) | 25 | 0.7186275 | 0.7494902 |
| Portugal Fish | -0.102 (0.114) | 18 | 0.8176275 | 0.8722745 |
| United Arab Emirates Birds | -0.099 (0.06) | 20 | 0.9189412 | 0.9490784 |
| Congo Mammals | -0.095 (0.046) | 14 | 0.9654510* | 0.9825490* |
| Ethiopia Mammals | -0.095 (0.087) | 21 | 0.8234510 | 0.8636078 |

Table S1: System Risks

| System | Theta (sd) | N | P(Theta) < 1.5 | P(Theta) < 0 |
|---|---|---|---|---|
| Brazil Mammals | -0.083 (0.079) | 142 | 0.8081961 | 0.8549020 |
| Brazil Birds | -0.081 (0.046) | 707 | 0.9271961 | 0.9623529* |
| Cte D'Ivoire Mammals | -0.081 (0.032) | 17 | 0.9829804* | 0.9940196* |
| Chile Birds | -0.08 (0.122) | 17 | 0.7062157 | 0.7474902 |
| Australia Mammals | -0.069 (0.035) | 1423 | 0.9408039 | 0.9766863* |
| Ecuador Birds | -0.068 (0.035) | 38 | 0.9382941 | 0.9749804* |
| Mexico Mammals | -0.066 (0.047) | 44 | 0.8595490 | 0.9190784 |
| Australia Birds | -0.065 (0.009) | 7064 | 1.0000000* | 1.0000000* |
| Chile Mammals | -0.062 (0.068) | 28 | 0.7543529 | 0.8181176 |
| Malaysia Herps | -0.061 (0.043) | 36 | 0.8592549 | 0.9231961 |
| French Polynesia Fish | -0.057 (0.094) | 29 | 0.6758627 | 0.7305098 |
| Namibia Birds | -0.057 (0.119) | 43 | 0.6380784 | 0.6849804 |
| Taiwan Fish | -0.054 (0.061) | 129 | 0.7387059 | 0.8118627 |
| Puerto Rico Birds | -0.05 (0.053) | 19 | 0.7466078 | 0.8291373 |
| Argentina Fish | -0.049 (0.034) | 52 | 0.8407255 | 0.9259804 |
| Cameroon Mammals | -0.048 (0.082) | 22 | 0.6593725 | 0.7249608 |
| Costa Rica Herps | -0.047 (0.069) | 26 | 0.6780980 | 0.7511961 |
| Mexico Herps | -0.044 (0.068) | 26 | 0.6638824 | 0.7457843 |
| Turkey Fish | -0.039 (0.034) | 12 | 0.7617451 | 0.8948431 |

Table S1: System Risks

| System | Theta (sd) | N | P(Theta) < 1.5 | P(Theta) < 0 |
|---|---|---|---|---|
| Mexico Fish | -0.037 (0.043) | 127 | 0.6925294 | 0.8063137 |
| France Herps | -0.033 (0.057) | 11 | 0.6277255 | 0.7234118 |
| China Mammals | -0.033 (0.088) | 35 | 0.5785490 | 0.6448431 |
| Romania Birds | -0.032 (0.051) | 37 | 0.6298431 | 0.7327843 |
| Seychelles Fish | -0.029 (0.085) | 18 | 0.5669020 | 0.6346863 |
| Italy Fish | -0.028 (0.035) | 75 | 0.6457451 | 0.7887843 |
| Sweden Herps | -0.027 (0.052) | 11 | 0.5873529 | 0.7000588 |
| Canada Mammals | -0.026 (0.019) | 567 | 0.7143725 | 0.9136667 |
| Haiti Fish | -0.026 (0.045) | 11 | 0.5928039 | 0.7156863 |
| New Zealand Fish | -0.025 (0.012) | 70 | 0.8028824 | 0.9821765* |
| Zimbabwe Mammals | -0.025 (0.042) | 49 | 0.5934706 | 0.7257255 |
| Madagascar Herps | -0.025 (0.054) | 31 | 0.5699412 | 0.6766667 |
| Finland Birds | -0.024 (0.018) | 102 | 0.6949216 | 0.9067451 |
| Japan Mammals | -0.024 (0.169) | 16 | 0.5201569 | 0.5562941 |
| Mexico Birds | -0.022 (0.036) | 80 | 0.5714706 | 0.7259804 |
| Puerto Rico Herps | -0.018 (0.046) | 32 | 0.5204902 | 0.6527059 |
| Australia Herps | -0.018 (0.025) | 126 | 0.5500392 | 0.7682353 |
| Argentina Mammals | -0.017 (0.079) | 20 | 0.5054118 | 0.5818431 |

Table S1: System Risks

| System | Theta (sd) | N | P(Theta) < 1.5 | P(Theta) < 0 |
|---|---|---|---|---|
| International Waters Fish | -0.016 (0.007) | 159 | 0.5529412 | 0.9850588* |
| Tanzania Mammals | -0.015 (0.02) | 178 | 0.5107451 | 0.7741961 |
| Peru Herps | -0.015 (0.092) | 30 | 0.4978039 | 0.5643333 |
| Chile Fish | -0.014 (0.033) | 42 | 0.4926078 | 0.6684902 |
| Sweden Fish | -0.013 (0.024) | 25 | 0.4737059 | 0.7063725 |
| South Georgia And The South Sandwich Islands Birds | -0.013 (0.023) | 27 | 0.4684902 | 0.7218824 |
| Cambodia Birds | -0.012 (0.036) | 22 | 0.4836078 | 0.6546078 |
| Korea Birds | -0.011 (0.06) | 64 | 0.4756863 | 0.5769608 |
| Malaysia Mammals | -0.011 (0.107) | 27 | 0.4872549 | 0.5426078 |
| Australia Fish | -0.011 (0.016) | 680 | 0.4044706 | 0.7602745 |
| Mauritania Birds | -0.011 (0.205) | 14 | 0.4796667 | 0.5116078 |
| Ghana Birds | -0.011 (0.205) | 30 | 0.4897451 | 0.5196078 |
| Sweden Mammals | -0.01 (0.067) | 55 | 0.4738431 | 0.5627647 |
| United States Herps | -0.01 (0.03) | 309 | 0.4385882 | 0.6345294 |
| India Mammals | -0.009 (0.024) | 94 | 0.4102353 | 0.6522353 |
| Norway Birds | -0.008 (0.012) | 372 | 0.2934314 | 0.7442353 |
| Canada Fish | -0.007 (0.007) | 3195 | 0.1120588 | 0.8524902 |
| Greece Fish | -0.007 (0.119) | 27 | 0.4710784 | 0.5206471 |
| Iran Birds | -0.006 (0.126) | 13 | 0.4695098 | 0.5172353 |
| Greece Herps | -0.005 (0.028) | 13 | 0.3583529 | 0.5667059 |

## Table S1: System Risks

| System | Theta (sd) | N | P(Theta) < 1.5 | P(Theta) < 0 |
|---|---|---|---|---|
| Kenya Mammals | -0.005 (0.033) | 135 | 0.3785490 | 0.5575098 |
| United States Fish | -0.005 (0.009) | 901 | 0.1153333 | 0.6992549 |
| Iceland Birds | -0.004 (0.01) | 34 | 0.1382745 | 0.6430000 |
| Switzerland Mammals | -0.003 (0.029) | 23 | 0.3383333 | 0.5415294 |
| Panama Herps | -0.002 (0.081) | 68 | 0.4283137 | 0.5035490 |
| South Africa Birds | -0.002 (0.037) | 142 | 0.3596863 | 0.5163529 |
| Northern Mariana Islands Birds | -0.002 (0.023) | 22 | 0.2726471 | 0.5270980 |
| Finland Mammals | -0.001 (0.037) | 83 | 0.3594510 | 0.5176078 |
| Russian Federation Mammals | -0.001 (0.032) | 91 | 0.3337647 | 0.5127647 |
| Ukraine Birds | -0.001 (0.035) | 136 | 0.3392353 | 0.5044510 |
| Canada Birds | 0 (0.001) | 2297 | 0.0000000 | 0.6180588 |
| Antarctica Birds | 0 (0.01) | 129 | 0.0574314 | 0.4809216 |
| Bangladesh Birds | 0.001 (0.296) | 20 | 0.4782353 | 0.4979412 |
| Japan Fish | 0.001 (0.023) | 41 | 0.2419412 | 0.4819216 |
| Spain Fish | 0.002 (0.051) | 106 | 0.3710980 | 0.4857059 |
| Argentina Birds | 0.002 (0.096) | 20 | 0.4278235 | 0.4876667 |
| Virgin Islands Fish | 0.002 (0.038) | 48 | 0.3229412 | 0.4726078 |
| Falkland Islands (Malvinas) Birds | 0.003 (0.014) | 83 | 0.1003137 | 0.4186667 |
| Belize Herps | 0.004 (0.089) | 29 | 0.4152549 | 0.4821373 |
| Singapore Birds | 0.004 (0.079) | 19 | 0.4037647 | 0.4795686 |
| United Kingdom Fish | 0.005 (0.015) | 143 | 0.0982157 | 0.3720196 |
| French Southern Territories Birds | 0.005 (0.014) | 33 | 0.0727255 | 0.3589020 |
| Iceland Fish | 0.005 (0.023) | 14 | 0.1812745 | 0.4049216 |
| Italy Herps | 0.007 (0.023) | 24 | 0.1704118 | 0.3901176 |

Table S1: System Risks

| System | Theta (sd) | N | P(Theta) < 1.5 | P(Theta) < 0 |
|---|---|---|---|---|
| Sweden Birds | 0.007 (0.014) | 156 | 0.0660196 | 0.3194706 |
| Indonesia Mammals | 0.007 (0.054) | 40 | 0.3442157 | 0.4525098 |
| Congo Herps | 0.007 (0.115) | 14 | 0.4245098 | 0.4825294 |
| Romania Mammals | 0.009 (0.011) | 12 | 0.0160196 | 0.2155098 |
| Zambia Fish | 0.009 (0.114) | 11 | 0.4128824 | 0.4665490 |
| Peru Birds | 0.009 (0.162) | 20 | 0.4394706 | 0.4749608 |
| Zambia Mammals | 0.011 (0.071) | 27 | 0.3570392 | 0.4404902 |
| Ecuador Mammals | 0.011 (0.089) | 16 | 0.3857059 | 0.4521765 |
| Estonia Birds | 0.012 (0.029) | 32 | 0.1733137 | 0.3342157 |
| Tanzania Fish | 0.012 (0.126) | 22 | 0.4279412 | 0.4781176 |
| Czechia Birds | 0.013 (0.02) | 23 | 0.0788824 | 0.2550000 |
| Poland Birds | 0.014 (0.018) | 68 | 0.0617647 | 0.2315294 |
| Turkey Birds | 0.014 (0.04) | 112 | 0.2398627 | 0.3670392 |
| United Kingdom Herps | 0.015 (0.106) | 22 | 0.3881961 | 0.4434510 |
| United Kingdom Birds | 0.015 (0.008) | 322 | 0.0000196 | 0.0201373 |
| Bulgaria Mammals | 0.016 (0.012) | 11 | 0.0103333 | 0.0854706 |
| Slovakia Birds | 0.016 (0.041) | 17 | 0.2244510 | 0.3478039 |
| Bulgaria Birds | 0.016 (0.042) | 39 | 0.2277059 | 0.3520392 |
| Netherlands Herps | 0.016 (0.049) | 16 | 0.2660392 | 0.3716078 |
| France Fish | 0.017 (0.05) | 87 | 0.2596078 | 0.3641765 |
| Czechia Fish | 0.018 (0.089) | 100 | 0.3562941 | 0.4176667 |
| Estonia Fish | 0.019 (0.087) | 13 | 0.3493922 | 0.4144706 |
| Morocco Birds | 0.019 (0.06) | 102 | 0.2844706 | 0.3748824 |
| Norway Mammals | 0.019 (0.081) | 34 | 0.3400588 | 0.4100980 |
| Greenland Birds | 0.021 (0.086) | 44 | 0.3376275 | 0.4018235 |
| United States Mammals | 0.021 (0.014) | 338 | 0.0057451 | 0.0667843 |
| Turkey Herps | 0.022 (0.041) | 31 | 0.1870392 | 0.2991765 |
| Canada Herps | 0.022 (0.039) | 149 | 0.1679216 | 0.2847059 |
| South Africa Mammals | 0.024 (0.019) | 111 | 0.0205294 | 0.1066078 |

Table S1: System Risks

| System | Theta (sd) | N | P(Theta) < 1.5 | P(Theta) < 0 |
|---|---|---|---|---|
| Hungary Birds | 0.024 (0.029) | 19 | 0.0860000 | 0.2014902 |
| Brazil Fish | 0.024 (0.025) | 2257 | 0.0570784 | 0.1623725 |
| Denmark Birds | 0.026 (0.023) | 93 | 0.0391176 | 0.1303137 |
| Seychelles Herps | 0.026 (0.047) | 14 | 0.1895294 | 0.2842353 |
| Peru Mammals | 0.027 (0.058) | 35 | 0.2364118 | 0.3235294 |
| Cyprus Herps | 0.028 (0.087) | 16 | 0.3126078 | 0.3767647 |
| Pakistan Mammals | 0.028 (0.022) | 13 | 0.0283137 | 0.1025490 |
| Russian Federation Birds | 0.028 (0.055) | 96 | 0.2160588 | 0.3028431 |
| China Birds | 0.029 (0.049) | 24 | 0.1832353 | 0.2788627 |
| Uganda Mammals | 0.029 (0.018) | 119 | 0.0078039 | 0.0561176 |
| Colombia Fish | 0.029 (0.073) | 22 | 0.2609020 | 0.3364902 |
| Japan Birds | 0.029 (0.032) | 22 | 0.0797647 | 0.1756471 |
| Netherlands Birds | 0.029 (0.013) | 44 | 0.0003529 | 0.0119216 |
| United States Birds | 0.03 (0.01) | 428 | 0.0000000 | 0.0006078 |
| Poland Mammals | 0.032 (0.02) | 76 | 0.0100588 | 0.0557843 |
| Germany Birds | 0.033 (0.014) | 213 | 0.0001765 | 0.0072549 |
| Uganda Birds | 0.033 (0.048) | 42 | 0.1559804 | 0.2421373 |
| Guinea Mammals | 0.035 (0.126) | 12 | 0.3327059 | 0.3800588 |
| New Zealand Birds | 0.035 (0.028) | 51 | 0.0389020 | 0.1083922 |
| Belarus Mammals | 0.036 (0.022) | 28 | 0.0094902 | 0.0471176 |
| South Africa Fish | 0.036 (0.035) | 298 | 0.0727255 | 0.1524706 |
| Lithuania Mammals | 0.037 (0.018) | 14 | 0.0025294 | 0.0190392 |
| Belgium Birds | 0.038 (0.055) | 12 | 0.1678627 | 0.2462745 |
| Austria Birds | 0.038 (0.049) | 33 | 0.1397255 | 0.2184902 |
| Ireland Birds | 0.038 (0.038) | 27 | 0.0766078 | 0.1496667 |
| Russian Federation Fish | 0.04 (0.053) | 36 | 0.1474706 | 0.2243333 |
| Slovakia Mammals | 0.041 (0.017) | 17 | 0.0004706 | 0.0058824 |
| Nepal Mammals | 0.041 (0.036) | 13 | 0.0570392 | 0.1202745 |
| Spain Birds | 0.042 (0.01) | 656 | 0.0000000 | 0.0000392 |

Table S1: System Risks

| System | Theta (sd) | N | P(Theta) < 1.5 | P(Theta) < 0 |
|---|---|---|---|---|
| United Kingdom Mammals | 0.043 (0.01) | 152 | 0.0000000 | 0.0000000 |
| Portugal Birds | 0.043 (0.04) | 32 | 0.0705490 | 0.1370784 |
| New Zealand Herps | 0.044 (0.055) | 19 | 0.1294118 | 0.2040392 |
| Ecuador Herps | 0.044 (0.157) | 14 | 0.3475294 | 0.3846275 |
| France Birds | 0.045 (0.009) | 280 | 0.0000000 | 0.0000000 |
| Italy Mammals | 0.046 (0.026) | 46 | 0.0112353 | 0.0418431 |
| Brazil Herps | 0.049 (0.056) | 76 | 0.1269608 | 0.1912745 |
| Spain Mammals | 0.049 (0.034) | 87 | 0.0293137 | 0.0746078 |
| Egypt Fish | 0.051 (0.034) | 11 | 0.0232157 | 0.0605098 |
| Zimbabwe Fish | 0.051 (0.079) | 50 | 0.1996471 | 0.2563137 |
| Greece Birds | 0.053 (0.065) | 54 | 0.1476471 | 0.2065490 |
| France Mammals | 0.055 (0.02) | 67 | 0.0001961 | 0.0025882 |
| Papua New Guinea Herps | 0.056 (0.086) | 21 | 0.2064118 | 0.2594902 |
| Madagascar Mammals | 0.056 (0.21) | 16 | 0.3629804 | 0.3922157 |
| Kenya Birds | 0.058 (0.141) | 20 | 0.3042941 | 0.3434314 |
| Mauritius Herps | 0.061 (0.031) | 11 | 0.0084706 | 0.0229216 |
| Costa Rica Mammals | 0.061 (0.079) | 33 | 0.1684902 | 0.2194314 |
| Suriname Herps | 0.063 (0.07) | 12 | 0.1294706 | 0.1805294 |
| Germany Mammals | 0.064 (0.034) | 28 | 0.0107255 | 0.0311176 |
| Switzerland Birds | 0.065 (0.041) | 14 | 0.0233922 | 0.0545098 |
| Senegal Birds | 0.067 (0.162) | 33 | 0.3077451 | 0.3378431 |
| Netherlands Mammals | 0.069 (0.016) | 48 | 0.0000000 | 0.0000588 |
| Norway Fish | 0.074 (0.028) | 153 | 0.0004706 | 0.0040000 |
| Bahamas Fish | 0.083 (0.038) | 274 | 0.0043725 | 0.0145294 |
| Antarctica Fish | 0.084 (0.121) | 13 | 0.2028431 | 0.2391569 |
| Denmark Mammals | 0.086 (0.047) | 12 | 0.0145490 | 0.0321569 |
| Botswana Birds | 0.089 (0.184) | 34 | 0.2857059 | 0.3151569 |
| Egypt Birds | 0.092 (0.238) | 14 | 0.3220784 | 0.3451961 |
| Italy Birds | 0.094 (0.022) | 139 | 0.0000000 | 0.0000000 |

## Table S1: System Risks

| System | Theta (sd) | N | P(Theta) < 1.5 | P(Theta) < 0 |
|---|---|---|---|---|
| India Herps | 0.106 (0.194) | 15 | 0.2651373 | 0.2906275 |
| Belgium Mammals | 0.11 (0.055) | 14 | 0.0177843 | 0.0291765 |
| Belize Fish | 0.121 (0.045) | 253 | 0.0011373 | 0.0034706 |
| Indonesia Herps | 0.123 (0.119) | 16 | 0.1199216 | 0.1478235 |
| Ireland Mammals | 0.166 (0.077) | 12 | 0.0068824 | 0.0112157 |
| Thailand Mammals | 0.218 (0.253) | 25 | 0.1777647 | 0.1932941 |
| Papua New Guinea Fish | 0.226 (0.228) | 14 | 0.1415098 | 0.1576471 |
| Cameroon Birds | 0.364 (0.244) | 25 | 0.0576275 | 0.0654706 |
| Mali Birds | 0.819 (0.56) | 14 | 0.0672157 | 0.0709412 |

## Table S2: System Specific Power Analyses for Detecting Improvement at 95% Confidence

| System | N = 128 | N = 256 | N = 1024 |
|---|---|---|---|
| Albania Birds | 0.635 | 0.725 | 0.911 |
| Argentina Fish | 0.213 | 0.206 | 0.123 |
| Argentina Mammals | 0.000 | 0.000 | 0.000 |
| Argentina Herps | 0.370 | 0.373 | 0.334 |
| Australia Birds | 0.191 | 0.288 | 0.689 |
| Australia Mammals | 0.176 | 0.239 | 0.433 |
| Australia Herps | 0.059 | 0.041 | 0.003 |
| Brazil Birds | 0.274 | 0.350 | 0.521 |
| Brazil Mammals | 0.077 | 0.044 | 0.001 |
| Cameroon Mammals | 0.000 | 0.000 | 0.000 |
| Canada Mammals | 0.120 | 0.144 | 0.177 |
| Central African Republic Mammals | 1.000 | 1.000 | 1.000 |
| Chile Birds | 0.000 | 0.000 | 0.000 |
| Chile Mammals | 0.025 | 0.005 | 0.000 |
| China Fish | 0.902 | 0.982 | 1.000 |

Table S2: System Specific Power Analyses for Detecting Improvement at 95% Confidence

| System | N = 128 | N = 256 | N = 1024 |
|---|---|---|---|
| China Mammals | 0.000 | 0.000 | 0.000 |
| Congo Mammals | 0.931 | 0.981 | 1.000 |
| Costa Rica Herps | 0.004 | 0.000 | 0.000 |
| Croatia Birds | 0.331 | 0.343 | 0.318 |
| Cte D'Ivoire Fish | 0.000 | 0.000 | 0.000 |
| Cte D'Ivoire Mammals | 0.999 | 1.000 | 1.000 |
| Ecuador Birds | 0.568 | 0.701 | 0.934 |
| Ethiopia Mammals | 0.044 | 0.014 | 0.000 |
| Finland Birds | 0.140 | 0.157 | 0.147 |
| France Herps | 0.000 | 0.000 | 0.000 |
| French Polynesia Fish | 0.000 | 0.000 | 0.000 |
| Germany Fish | 0.515 | 0.610 | 0.820 |
| Ghana Birds | 0.000 | 0.000 | 0.000 |
| Guam Birds | 0.990 | 1.000 | 1.000 |
| Haiti Fish | 0.000 | 0.000 | 0.000 |
| Iceland Mammals | 0.128 | 0.074 | 0.005 |
| India Birds | 1.000 | 1.000 | 1.000 |
| International Waters Fish | 0.216 | 0.302 | 0.566 |
| Italy Fish | 0.042 | 0.017 | 0.000 |
| Japan Mammals | 0.000 | 0.000 | 0.000 |
| Madagascar Herps | 0.000 | 0.000 | 0.000 |
| Malaysia Herps | 0.197 | 0.171 | 0.066 |
| Mexico Fish | 0.003 | 0.000 | 0.000 |
| Mexico Birds | 0.022 | 0.006 | 0.000 |
| Mexico Mammals | 0.175 | 0.140 | 0.036 |
| Mexico Herps | 0.005 | 0.000 | 0.000 |
| Namibia Birds | 0.000 | 0.000 | 0.000 |
| New Zealand Fish | 0.356 | 0.482 | 0.777 |

Table S2: System Specific Power Analyses for Detecting Improvement at 95% Confidence

| System | N = 128 | N = 256 | N = 1024 |
|---|---|---|---|
| Nigeria Birds | 0.044 | 0.014 | 0.000 |
| Pakistan Birds | 1.000 | 1.000 | 1.000 |
| Panama Mammals | 0.316 | 0.306 | 0.227 |
| Peru Herps | 0.000 | 0.000 | 0.000 |
| Philippines Fish | 0.111 | 0.059 | 0.001 |
| Portugal Fish | 0.003 | 0.001 | 0.000 |
| Portugal Herps | 0.998 | 1.000 | 1.000 |
| Puerto Rico Fish | 0.124 | 0.072 | 0.003 |
| Puerto Rico Birds | 0.025 | 0.005 | 0.000 |
| Puerto Rico Herps | 0.000 | 0.000 | 0.000 |
| Romania Fish | 0.997 | 1.000 | 1.000 |
| Romania Birds | 0.017 | 0.003 | 0.000 |
| Seychelles Fish | 0.000 | 0.000 | 0.000 |
| Sweden Herps | 0.000 | 0.000 | 0.000 |
| Taiwan Fish | 0.081 | 0.073 | 0.026 |
| Tanzania Mammals | 0.056 | 0.035 | 0.001 |
| Turkey Fish | 0.024 | 0.005 | 0.000 |
| United Arab Emirates Birds | 0.378 | 0.410 | 0.447 |
| Zimbabwe Mammals | 0.026 | 0.008 | 0.000 |

Table S3: System Specific Power Analyses for Identifying Current Declines

| System | N = 128 | N = 256 | N = 1024 |
|---|---|---|---|
| Albania Birds | 0.927 | 0.935 | 0.945 |
| Argentina Fish | 0.745 | 0.793 | 0.857 |
| Argentina Mammals | 0.397 | 0.450 | 0.516 |
| Argentina Herps | 0.903 | 0.920 | 0.938 |

Table S3: System Specific Power Analyses for Identifying Current Declines

| System | N = 128 | N = 256 | N = 1024 |
|---|---|---|---|
| Australia Birds | 1.000 | 1.000 | 1.000 |
| Australia Mammals | 0.881 | 0.865 | 0.885 |
| Australia Herps | 0.224 | 0.351 | 0.547 |
| Brazil Birds | 0.801 | 0.838 | 0.900 |
| Brazil Mammals | 0.600 | 0.678 | 0.775 |
| Cameroon Mammals | 0.548 | 0.594 | 0.647 |
| Canada Mammals | 0.390 | 0.499 | 0.678 |
| Central African Republic Mammals | 0.991 | 0.991 | 0.992 |
| Chile Birds | 0.595 | 0.645 | 0.702 |
| Chile Mammals | 0.588 | 0.654 | 0.736 |
| China Fish | 0.972 | 0.979 | 0.986 |
| China Mammals | 0.439 | 0.496 | 0.564 |
| Congo Mammals | 0.956 | 0.964 | 0.973 |
| Costa Rica Herps | 0.503 | 0.579 | 0.670 |
| Croatia Birds | 0.849 | 0.877 | 0.912 |
| Cte D'Ivoire Fish | 0.588 | 0.639 | 0.697 |
| Cte D'Ivoire Mammals | 0.982 | 0.984 | 0.987 |
| Ecuador Birds | 0.913 | 0.933 | 0.957 |
| Ethiopia Mammals | 0.714 | 0.761 | 0.815 |
| Finland Birds | 0.499 | 0.605 | 0.758 |
| France Herps | 0.550 | 0.604 | 0.667 |
| French Polynesia Fish | 0.589 | 0.630 | 0.676 |
| Germany Fish | 0.892 | 0.915 | 0.943 |
| Ghana Birds | 0.335 | 0.386 | 0.450 |
| Guam Birds | 0.998 | 0.998 | 0.998 |
| Haiti Fish | 0.603 | 0.638 | 0.678 |
| Iceland Mammals | 0.856 | 0.878 | 0.901 |
| India Birds | 1.000 | 1.000 | 1.000 |
| International Waters Fish | 0.945 | 0.928 | 0.936 |

Table S3: System Specific Power Analyses for Identifying Current Declines

| System | N = 128 | N = 256 | N = 1024 |
|---|---|---|---|
| Italy Fish | 0.425 | 0.525 | 0.654 |
| Japan Mammals | 0.365 | 0.425 | 0.500 |
| Madagascar Herps | 0.392 | 0.469 | 0.565 |
| Malaysia Herps | 0.800 | 0.842 | 0.890 |
| Mexico Fish | 0.646 | 0.694 | 0.751 |
| Mexico Birds | 0.326 | 0.431 | 0.574 |
| Mexico Mammals | 0.789 | 0.831 | 0.883 |
| Mexico Herps | 0.479 | 0.555 | 0.653 |
| Namibia Birds | 0.417 | 0.489 | 0.582 |
| New Zealand Fish | 0.896 | 0.905 | 0.935 |
| Nigeria Birds | 0.864 | 0.884 | 0.903 |
| Pakistan Birds | 1.000 | 1.000 | 1.000 |
| Panama Mammals | 0.885 | 0.904 | 0.925 |
| Peru Herps | 0.414 | 0.458 | 0.511 |
| Philippines Fish | 0.815 | 0.844 | 0.872 |
| Portugal Fish | 0.763 | 0.799 | 0.838 |
| Portugal Herps | 0.990 | 0.991 | 0.993 |
| Puerto Rico Fish | 0.830 | 0.858 | 0.888 |
| Puerto Rico Birds | 0.621 | 0.681 | 0.753 |
| Puerto Rico Herps | 0.397 | 0.469 | 0.559 |
| Romania Fish | 0.982 | 0.985 | 0.989 |
| Romania Birds | 0.390 | 0.491 | 0.625 |
| Seychelles Fish | 0.470 | 0.517 | 0.575 |
| Sweden Herps | 0.519 | 0.577 | 0.642 |
| Taiwan Fish | 0.208 | 0.341 | 0.566 |
| Tanzania Mammals | 0.300 | 0.425 | 0.600 |
| Turkey Fish | 0.776 | 0.818 | 0.864 |
| United Arab Emirates Birds | 0.882 | 0.904 | 0.929 |
| Zimbabwe Mammals | 0.307 | 0.421 | 0.575 |

Table S4: System Specific Power Analyses Under Modified Thresholds

| | Fixed "reference" threshold 1.5%/yr | | | Detecting Changes at 70% Confidence | | |
|---|---|---|---|---|---|---|
| **System** | **N = 128** | **N = 256** | **N = 1024** | **N = 128** | **N = 256** | **N = 1024** |
| Albania Birds | 0.116 | 0.158 | 0.361 | 1.000 | 1.000 | 1.000 |
| Argentina Fish | 0.188 | 0.286 | 0.697 | 0.926 | 0.985 | 1.000 |
| Argentina Mammals | 0.160 | 0.234 | 0.578 | 0.124 | 0.057 | 0.001 |
| Argentina Herps | 0.082 | 0.100 | 0.182 | 1.000 | 1.000 | 1.000 |
| Australia Birds | 0.071 | 0.082 | 0.128 | 0.602 | 0.719 | 0.954 |
| Australia Mammals | 0.071 | 0.083 | 0.133 | 0.615 | 0.730 | 0.948 |
| Australia Herps | 0.132 | 0.186 | 0.446 | 0.477 | 0.528 | 0.658 |
| Brazil Birds | 0.088 | 0.110 | 0.212 | 0.822 | 0.927 | 1.000 |
| Brazil Mammals | 0.090 | 0.113 | 0.221 | 0.765 | 0.869 | 0.993 |
| Cameroon Mammals | 0.171 | 0.255 | 0.627 | 0.568 | 0.613 | 0.742 |
| Canada Mammals | 0.103 | 0.136 | 0.293 | 0.533 | 0.619 | 0.839 |
| Central African Republic Mammals | 0.322 | 0.502 | 0.916 | 1.000 | 1.000 | 1.000 |
| Chile Birds | 0.110 | 0.146 | 0.325 | 0.648 | 0.722 | 0.895 |
| Chile Mammals | 0.118 | 0.161 | 0.372 | 0.758 | 0.858 | 0.989 |
| China Fish | 0.152 | 0.221 | 0.544 | 1.000 | 1.000 | 1.000 |
| China Mammals | 0.136 | 0.193 | 0.467 | 0.282 | 0.224 | 0.076 |
| Congo Mammals | 0.420 | 0.636 | 0.960 | 1.000 | 1.000 | 1.000 |
| Costa Rica Herps | 0.125 | 0.173 | 0.406 | 0.597 | 0.664 | 0.833 |
| Croatia Birds | 0.079 | 0.095 | 0.166 | 0.997 | 1.000 | 1.000 |
| Cte D'Ivoire Fish | 0.087 | 0.108 | 0.207 | 0.650 | 0.726 | 0.901 |
| Cte D'Ivoire Mammals | 0.579 | 0.825 | 0.997 | 1.000 | 1.000 | 1.000 |
| Ecuador Birds | 0.199 | 0.304 | 0.734 | 0.996 | 1.000 | 1.000 |
| Ethiopia Mammals | 0.112 | 0.150 | 0.337 | 0.909 | 0.975 | 1.000 |
| Finland Birds | 0.143 | 0.206 | 0.502 | 0.619 | 0.724 | 0.933 |
| France Herps | 0.200 | 0.306 | 0.735 | 0.540 | 0.579 | 0.688 |
| French Polynesia Fish | 0.161 | 0.237 | 0.583 | 0.605 | 0.664 | 0.818 |

Table S4: System Specific Power Analyses Under Modified Thresholds

| | Fixed "reference" threshold 1.5%/yr | | | Detecting Changes at 70% Confidence | | |
|---|---|---|---|---|---|---|
| **System** | **N = 128** | **N = 256** | **N = 1024** | **N = 128** | **N = 256** | **N = 1024** |
| Germany Fish | 0.114 | 0.155 | 0.351 | 0.999 | 1.000 | 1.000 |
| Ghana Birds | 0.084 | 0.103 | 0.191 | 0.033 | 0.005 | 0.000 |
| Guam Birds | 0.105 | 0.138 | 0.300 | 1.000 | 1.000 | 1.000 |
| Haiti Fish | 0.518 | 0.763 | 0.992 | 0.563 | 0.602 | 0.717 |
| Iceland Mammals | 0.096 | 0.123 | 0.252 | 0.998 | 1.000 | 1.000 |
| India Birds | 0.085 | 0.105 | 0.197 | 1.000 | 1.000 | 1.000 |
| International Waters Fish | 0.251 | 0.396 | 0.870 | 0.677 | 0.800 | 0.982 |
| Italy Fish | 0.149 | 0.216 | 0.531 | 0.587 | 0.664 | 0.849 |
| Japan Mammals | 0.086 | 0.106 | 0.200 | 0.095 | 0.036 | 0.000 |
| Madagascar Herps | 0.148 | 0.215 | 0.528 | 0.393 | 0.380 | 0.312 |
| Malaysia Herps | 0.186 | 0.281 | 0.687 | 0.965 | 0.997 | 1.000 |
| Mexico Fish | 0.281 | 0.445 | 0.908 | 0.823 | 0.914 | 0.998 |
| Mexico Birds | 0.146 | 0.209 | 0.513 | 0.479 | 0.515 | 0.603 |
| Mexico Mammals | 0.176 | 0.264 | 0.650 | 0.968 | 0.998 | 1.000 |
| Mexico Herps | 0.124 | 0.171 | 0.402 | 0.562 | 0.617 | 0.763 |
| Namibia Birds | 0.088 | 0.109 | 0.209 | 0.414 | 0.408 | 0.364 |
| New Zealand Fish | 0.281 | 0.446 | 0.916 | 0.868 | 0.959 | 1.000 |
| Nigeria Birds | 0.082 | 0.099 | 0.180 | 0.999 | 1.000 | 1.000 |
| Pakistan Birds | 0.095 | 0.121 | 0.247 | 1.000 | 1.000 | 1.000 |
| Panama Mammals | 0.112 | 0.151 | 0.338 | 0.998 | 1.000 | 1.000 |
| Peru Herps | 0.168 | 0.249 | 0.615 | 0.057 | 0.014 | 0.000 |
| Philippines Fish | 0.097 | 0.125 | 0.258 | 0.998 | 1.000 | 1.000 |
| Portugal Fish | 0.220 | 0.339 | 0.709 | 0.954 | 0.979 | 0.997 |
| Portugal Herps | 0.107 | 0.142 | 0.309 | 1.000 | 1.000 | 1.000 |
| Puerto Rico Fish | 0.095 | 0.121 | 0.246 | 0.997 | 1.000 | 1.000 |
| Puerto Rico Birds | 0.158 | 0.231 | 0.570 | 0.801 | 0.899 | 0.997 |
| Puerto Rico Herps | 0.201 | 0.309 | 0.738 | 0.340 | 0.305 | 0.181 |

Table S4: System Specific Power Analyses Under Modified Thresholds

| | Fixed “reference” threshold 1.5%/yr | | | Detecting Changes at 70% Confidence | | |
|---|---|---|---|---|---|---|
| **System** | **N = 128** | **N = 256** | **N = 1024** | **N = 128** | **N = 256** | **N = 1024** |
| Romania Fish | 0.067 | 0.076 | 0.114 | 1.000 | 1.000 | 1.000 |
| Romania Birds | 0.116 | 0.158 | 0.361 | 0.499 | 0.540 | 0.644 |
| Seychelles Fish | 0.168 | 0.249 | 0.614 | 0.218 | 0.146 | 0.021 |
| Sweden Herps | 0.236 | 0.369 | 0.829 | 0.458 | 0.463 | 0.460 |
| Taiwan Fish | 0.069 | 0.079 | 0.122 | 0.491 | 0.555 | 0.722 |
| Tanzania Mammals | 0.186 | 0.280 | 0.686 | 0.510 | 0.568 | 0.721 |
| Turkey Fish | 0.446 | 0.679 | 0.980 | 0.972 | 0.995 | 1.000 |
| United Arab Emirates Birds | 0.167 | 0.247 | 0.600 | 0.996 | 1.000 | 1.000 |
| Zimbabwe Mammals | 0.118 | 0.161 | 0.371 | 0.466 | 0.499 | 0.578 |
